# Fine-Tuning Models for Histopathological Classification of Colorectal Cancer

**DOI:** 10.3390/diagnostics15151947

**Published:** 2025-08-03

**Authors:** Houda Saif ALGhafri, Chia S. Lim

**Affiliations:** 1 Department of Information Technology, College of Computing and Information Sciences, University of Technology and Applied Sciences, Muscat 133, Oman; 2 Graduate School of Technology, Asia Pacific University of Technology and Innovation, Kuala Lumpur 57000, Malaysia; lim.chiasien@apu.edu.my

**Keywords:** deep learning, transfer learning, fine-tuning, colorectal cancer, histopathological images

## Abstract

**Background/Objectives:** This study aims to design and evaluate transfer learning strategies that fine-tune multiple pre-trained convolutional neural network architectures based on their characteristics to improve the accuracy and generalizability of colorectal cancer histopathological image classification. **Methods:** The application of transfer learning with pre-trained models on specialized and multiple datasets is proposed, where the proposed models, CRCHistoDense, CRCHistoIncep, and CRCHistoXcep, are algorithmically fine-tuned at varying depths to improve the performance of colorectal cancer classification. These models were applied to datasets of 10,613 images from public and private repositories, external sources, and unseen data. To validate the models’ decision-making and improve transparency, we integrated Grad-CAM to provide visual explanations that influence classification decisions. **Results and Conclusions:** On average across all datasets, CRCHistoDense, CRCHistoIncep, and CRCHistoXcep achieved test accuracies of 99.34%, 99.48%, and 99.45%, respectively, highlighting the effectiveness of fine-tuning in improving classification performance and generalization. Statistical methods, including paired *t*-tests, ANOVA, and the Kruskal–Wallis test, confirmed significant improvements in the proposed methods’ performance, with *p*-values below 0.05. These findings demonstrate that fine-tuning based on the characteristics of CNN’s architecture enhances colorectal cancer classification in histopathology, thereby improving the diagnostic potential of deep learning models.

## 1. Introduction

Colorectal cancer (CRC) is an intestinal cancer that begins as polyps and gradually develops into malignant cells, leading to high incidence and mortality rates over time [1]. The stage at which CRC is diagnosed is crucial in determining survival outcomes. The American Joint Committee on Cancer (AJCC) TNM system is the standard staging framework for CRC, assessing three key factors: tumor (T), which evaluates the depth of cancer invasion into the colon or rectal walls; lymph nodes (N), which assesses whether cancer has spread to nearby lymph nodes; and metastasis (M), which identifies the presence of cancer in distant lymph nodes or organs like the liver or lungs [2,3,4]. This staging system is crucial for informing treatment decisions and predicting patient outcomes. Pathology slides for cancer diagnosis are routinely prepared from biopsy samples by staining tumor tissue with hematoxylin and eosin [5]. Histopathological analysis remains the most reliable method for diagnosing malignant tumors and various diseases [6]. However, this process is labor-intensive and time-consuming and demands high-level expertise, making it challenging for pathologists [7]. The complexity of analyzing histopathological images can lead to pathologist fatigue, increasing the risk of diagnostic errors [8]. Improving the efficiency and accuracy of histopathological assessment is, therefore, a critical need, particularly in the context of CRC diagnostics.

Digital pathology converts histology into multi-gigapixel whole slide images (WSIs), often reaching several gigabytes in size. Due to their large scale, loading WSIs entirely into memory for training machine learning models presents a considerable challenge [9]. To overcome such a challenge, WSIs are typically segmented into smaller image patches, as full-image analysis is computationally impractical [10,11,12,13]. A widely adopted approach involves dividing these large images into patches and applying deep-learning models to analyze each patch individually [10]. Such methods enable computational processing and facilitate automatic histopathological image classification. By leveraging deep learning-based classification, this approach enhances diagnostic efficiency by rapidly distinguishing between malignant and benign tissues, thereby improving patient outcomes [14].

Among the deep learning-based methods, convolutional neural networks (CNNs) have become a standard method for pathological image analysis due to their effectiveness in image classification [15]. Architectures such as Inception V3, Xception, and DenseNet have demonstrated strong performance in medical imaging tasks. However, annotating medical images is expensive and time-consuming, leading to limited labeled datasets [16]. To deal with such limitations, transfer learning techniques are extensively applied in medical image analysis [16]. Usually, models are initialized with weights pre-trained on the ImageNet dataset [17] and then fine-tuned using histopathological images [18]. That approach enables pre-trained models to capture distinct features relevant to the task by allowing their layers to learn specific characteristics essential for the accurate classification of CRC. According to [19], fine-tuning pre-trained networks can be performed using two primary methods: layer-wise fine-tuning and partial training. In layer-wise fine-tuning, individual layers are trained sequentially, with careful selection of which ones remain fixed and which ones undergo training. In contrast, partial training keeps the early layers frozen while training only the higher layers on the new dataset. The choice of layers to fine-tune is critical for the final classification performance of the proposed models, as it directly affects their capacity to extract relevant features from CRC histopathological images.

The objective of this study is to enhance the performance and generalizability of CRC histopathological image classification by providing specific fine-tuning methods on pre-trained CNN models through transfer learning. Specifically, we investigate how different fine-tuning strategies influence model adaptability and diagnostic accuracy across multiple CRC datasets. This research is motivated by the need for deep learning models that can perform reliably across diverse histopathological datasets, despite the challenges posed by limited annotations and morphological variability in CRC tissue samples. To address this, we proposed CRCHisto (Colorectal Cancer Histopathology) models based on three widely used CNN architectures: DenseNet121 (CRCHistoDense), InceptionV3 (CRCHistoIncep), and Xception (CRCHistoXcep). The explicit method of fine-tuning them on CRC histopathological images will provide a reference for other CNN models. The selection of these models was based on their complementary strengths: feature preservation, multi-scale processing, and efficiency in capturing complex image structures. These models were fine-tuned and evaluated using public, private, and integrated CRC datasets to ensure robustness. Furthermore, we investigated the impact of depth fine-tuning and feature randomization on performance and evaluated model generalization on internal, external, and unseen test sets.

The key contributions of our study are as follows: (1) A structured fine-tuning approach was applied to multiple pre-trained models (DenseNet121, InceptionV3, Xception), revealing performance dynamics specific to CRC histopathological image classification; (2) an analysis across internal, external, and unseen datasets revealed that fine-tuning enhanced the classification performance of CRC histopathological image models, with consistent improvements observed across all dataset sources; (3) we measured how different random initializations impact model performance by running multiple experiments, highlighting variability and ensuring reproducibility; (4) a comparative evaluation between baseline and fine-tuned models showed consistent and statistically significant improvements in CRC histopathological image classification; (5) a clinically curated dataset was developed to reflect CRC progression, incorporating key tissue types, adipose, muscle, and lymph nodes, to enhance the proposed models’ diagnostic relevance.

## 2. Related Works

Deep learning models excel in various medical imaging tasks because they automatically extract and learn intricate, hidden patterns within medical images, as shown by Ijaz et al. [20], enabling more accurate and efficient analysis. Transfer learning with pre-trained models has demonstrated potential in accurately classifying CRC tissue images. As a result, this section explores methodologies that employ deep learning techniques, with a focus on transfer learning strategies using CNNs. By examining recent studies, we highlight how these architectures have been effectively applied to CRC image classification, demonstrating advancements in leveraging pre-trained models to enhance diagnostic accuracy.

In the study by Tsai and Tao [21], CRC histopathological image classification was enhanced by utilizing transfer learning with pre-trained models, including AlexNet, SqueezeNet, VGGNet, GoogLeNet, and ResNet50. They used two public datasets, 5000 CRC images and 100,000 images, along with 7180 external validation images. The data were split into training (70%), testing (15%), and validation (15%) sets. ResNet50 achieved a classification accuracy of 94.86%. Likewise, Al. Shawesh and Chen [22] employed 100,000 CRC histopathological images for training and 7180 for validation. Using transfer learning with fine-tuning, they applied the ResNet50 model, initializing it with parameters trained on the ImageNet dataset and freezing all layers except the final ones. The model achieved a validation accuracy of 97.7%. Vidyun et al. [23] used transfer learning to fine-tune the VGG19 architecture, training only the top five layers while keeping the remaining 11 layers unchanged. They applied this approach to a public CRC histology image dataset of 5000 images, each annotated into eight classes and resized to 150 × 150 pixels. The fine-tuned model achieved an accuracy of 91.2%. Sarwinda et al. [24] utilized a public dataset of 165 benign and malignant tumor images, applying transfer learning with ResNet18 and ResNet50 architectures for classification. ResNet50 outperformed ResNet18, achieving accuracy rates between 73% and 88%, with sensitivity values ranging from 64% to 96%. Tasnim et al. [25] used the MobileNetV2 model on a public dataset of colon tissue images, achieving 99.67% accuracy with an 80/20 train–test split.

Several studies have investigated customized CNN architectures for the classification of CRC histopathological images. For example, Ibrahim et al. [26] developed a CNN model for classifying CRC images using a public dataset of 2500 images resized to 64 × 64 pixels. The architecture consisted of two convolutional layers with 3 × 3 filters and a fully connected layer with softmax activation, achieving an accuracy of 83%. In Kumar et al. [27], they explored multiple CNN architectures on a public dataset of normal colon and colon adenocarcinoma images. The top-performing model reached an accuracy of 99.40%. The dataset used in this study may present limitations due to its lack of diversity. The dataset primarily consists of normal colon and colon adenocarcinoma images, which may limit the model’s ability to generalize to variations commonly encountered in clinical practice.

Transfer learning using ResNet152, ResNet50, and VGG16 architectures was employed in the study by Naga Raju and Rao [28] to classify CRC images from a publicly available dataset of 5000 images. The dataset was split into 60% training, 30% testing, and 10% validation. The metrics indicated that ResNet152 delivered the highest accuracy at 98.38%, followed closely by ResNet50 at 97.08% and VGG16 at 96.16%. To classify colorectal cancer categories, Gupta et al. [29] utilized transfer learning with five pre-trained CNN architectures: ResNet50, Inception V3, VGG16, VGG19, and ResNet152V2. They experimented with a publicly available dataset of 5000 images, split into 70% training, 15% validation, and 15% testing. Inception V3 outperformed the other models, achieving the highest accuracy of 89.87%. This demonstrates the potential of transfer learning in CRC classification, though the relatively low accuracy suggests limitations in the dataset’s diversity and the potential for further improvement in model performance.

Transfer learning with fine-tuned VGG16 and MobileNetV2 has been used by Parelanickal et al. [30] for colon cancer tissue classification, utilizing an open-source dataset of 7200 histopathological images categorized into nine classes. The dataset was divided into 60% training, 20% testing, and 20% validation. MobileNetV2 achieved 97% accuracy, while VGG16 achieved 95%. Abhishek et al. [31] applied transfer learning with ResNet34 and EfficientNetB4 to classify CRC using a public dataset of 5000 histopathological images across eight tissue classes. ResNet34 achieved an accuracy of 99.97%, while EfficientNetB4 reached 99.8%. Despite the high accuracy, using a single public dataset raises concerns about dataset diversity, which could limit the model’s generalizability to other actual settings. Furthermore, as highlighted by Davila et al. [32], fine-tuning in medical image classification remains a challenging task, requiring careful model selection to balance complexity, accuracy, data availability, and computational efficiency.

Although notable progress has been made in CRC classification using deep learning, several critical gaps remain unaddressed in the literature. First, most studies lack rigorous statistical validation [22,23,27], often reporting only accuracy without confirming significance across experimental variations. Second, dataset bias remains a persistent issue, as public datasets offer limited diversity, thereby restricting model generalizability. Third, in studies such as [24,26,28,31], there is an insufficient methodological exploration of fine-tuning in relation to how depth and characteristics of architecture-specific tuning impact model learning, an aspect that can provide more reliability in fine-tuning other CNN models at large. Fourth, model interpretability is frequently absent or insufficient, which limits trust in real-world clinical settings. To address these gaps, our study investigates the impact of transfer learning and fine-tuning techniques on improving CRC histopathological image classification. The contributions made by this work are significant. We established a structured fine-tuning process, which led to consistent and statistically significant performance improvements across various datasets, including internal, external, and unseen datasets. Additionally, a clinically curated dataset was developed, reflecting the progression of CRC through the inclusion of key tissue types, further enhancing the relevance of the models. Visualization was integrated to improve the interpretability of the proposed models, facilitating an understanding of the features that drive classification decisions. These advancements strengthen the potential for refining deep learning models to improve medical diagnostics in CRC.

## 3. Dataset and Preprocessing

We utilized diverse datasets, with the primary input features consisting of RGB image patches extracted from colorectal histopathology slides. These patches served as the foundational data for model training and evaluation, capturing tissue-level characteristics critical for accurate classification. To ensure clinical relevance, the datasets were curated in consultation with the Pathology Department, focusing on representative tissue types associated with CRC progression, specifically adipose, muscle, and lymph node. The curation strategy was uniformly applied across all datasets, including private, public, and integrated sources, to enhance the generalizability and real-world applicability of the proposed models.

**Figure 1:**
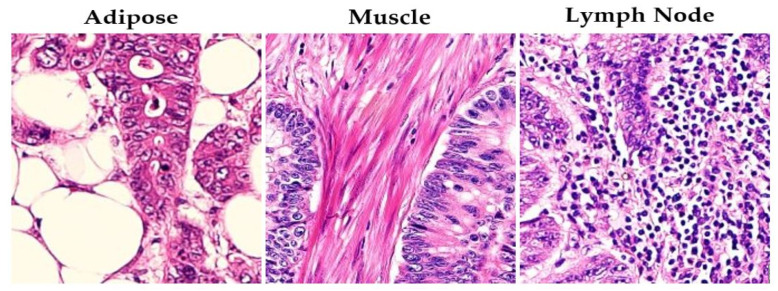
Representative samples of adipose tissue, muscle, and lymph node from Dataset 1, highlighting the morphological diversity relevant to CRC progression. These variations are critical for training robust classification models, as they reflect real-world variability that the model must generalize across. This visual context supports the inclusion of domain-representative features in our fine-tuning process.

The datasets include Dataset 1 (private source: Pathology Department, the Royal Hospital, the Sultanate of Oman), Dataset 2 (public source: [33]), and Dataset 3 (a combination of public and private sources) obtained from three public datasets, “CRC-VAL-HE-7K”, “NCT-CRC-HE-100K”, and “Colorectal Histology MNIST” [33,34], and the private dataset. Figure 1 illustrates adipose, muscle, and lymph node samples from Dataset 1, while Table 1 presents the distribution of CRC histopathological images across all used datasets.

Hematoxylin and eosin dyes, which are commonly used for staining tissue samples, aiding pathologists in the histopathological analysis essential for accurate CRC diagnosis [35], were also applied in this study. As part of the data collection process, we employed preprocessing methods to improve the quality and usability of these stained images. This included applying preprocessing steps to all datasets, such as resizing images to 224 × 224 pixels and normalizing pixel values for compatibility with pre-trained models. The private dataset consisted of stained histopathological images scanned at magnifications of 20×, 40×, and, in a few cases, 60×, depending on the tissue slide. The original images were captured at a resolution of 1920 × 1080 pixels. During preprocessing, WSIs containing artifacts were excluded to ensure the quality of the data. Representative tissue regions were manually selected using QuPath software [36], version 0.5.0, and then cropped into fixed-size patches of 224 × 224 pixels to ensure consistency and compatibility across datasets for learning and performance validation. The data was allocated as 65% for training, 15% for validation, and 20% for testing. These steps were designed to improve computational efficiency, focus on critical regions, and enhance the accuracy and reliability of CRC histopathological image classification.

## 4. Transfer Learning with Fine-Tuning Approach

Transfer learning is a versatile approach in deep learning [32] that leverages features from a pre-trained model to address related tasks more effectively, speeding up the training process on new data using previously acquired information. This concept [37] was initially introduced for image classification, laying the foundation for its widespread application. Accordingly, it is inspired by humans’ capability to transfer knowledge across domains, utilizing a related source domain to enhance learning or reduce labeled data in a target domain [38]. Our approach focuses on feature extraction without fine-tuning initially, adapting pre-trained models for CRC classification. In addition, we propose a method leveraging layer-wise and partial fine-tuning to enhance the reliability of fine-tuning for classifying CRC histopathological images across different pre-trained models. For example, layer-wise fine-tuning is employed when higher-level features necessitate fine-grained adaptation to CRC-specific patterns, allowing for selective layer updates that strike a balance between generalization and learning the current task. Partial fine-tuning is implemented because of limited computational resources, allowing updates to only a subset of layers. This strategic variation ensures that the fine-tuning process is optimized for the strengths and limitations of each pre-trained model. Furthermore, fine-tuning is closely related to hyperparameter optimization due to the growing complexity of deep learning architectures [39]. As models become deeper, selecting the correct layers to fine-tune along with tuning hyperparameters, such as learning rate and batch size, become crucial for achieving acceptable performance. Based on the state-of-the-art CNNs and the factors considered above, we selected DenseNet121, Inception V3, and Xception to enhance CRC classification using histopathological images. DenseNet, introduced by [40], builds on ResNet’s principles with densely connected layers, enhancing information flow, gradient propagation, and feature reuse while minimizing information loss. Inception V3, introduced by [41], optimizes CNN efficiency, offering improved performance at a computational cost 2.5 times lower than that of GoogLeNet and significantly more efficient than VGGNet. Xception, proposed by [42], enhances the Inception module by replacing it with depthwise separable convolutions, followed by pointwise convolutions, forming a linear stack with residual connections to improve efficiency and performance.

## 5. Experimental Setup

In our experiment, the training process began with pre-trained models as the baseline, followed by fine-tuning on the CRC datasets presented in Table 1. The proposed models were developed and trained using the TensorFlow Keras library with Python version 3.10.13. All experiments were conducted on a Dell laptop equipped with a 13th-generation Intel Core i9-11390HX processor and an NVIDIA GeForce RTX 4060 GPU, sourced in Muscat, Oman. We initially evaluated the proposed models on various datasets, assessing their performance using multiple metrics, as outlined in Equations (1)–(7). In addition, each model was applied to the integrated dataset, which combines diverse CRC histopathological images, to evaluate the impact of depth fine-tuning with various feature initializations to ensure robust and reliable results. Furthermore, statistical significance tests were used to assess the hypothesis of depth fine-tuning on the performance of the proposed models. The statistical tests employed repeated ANOVA with 95% confidence intervals and the Shapiro–Wilk test [43] to assess the normality of the data distribution. These tests evaluated the reliability of the results across different runs with varying initializations. In addition, validation was performed on external sources and unseen data to assess the models’ generalization capability.

(1) $$Accuracy=\frac{True\;Positive+True\;Negative}{True\;Positive+True\;Negative+False\;Negative+False\;Positive}$$

(2) $$Sensitivity\left(Recall\right)=\frac{True\;Positive}{True\;Positive+False\;Negative}$$

(3) $$Specificity=\frac{True\;Negative}{True\;Negative+False\;Positive}$$

(4) $$Precision=\frac{True\;Positive}{True\;Positive+False\;Positive}$$

(5) $$F1\;score=\frac{2\times Precision\times Sensitivity}{Precision+Sensitivity}$$

(6) $$Misclassification\;Rate=\frac{Number\;of\;misclassified\;samples}{\left(Total\;number\;of\;samples\right)}\;\times 100$$

(7) $$kappa=\frac{P_{o}-P_{e}}{1-P_{e}}\;$$

$P_{o}$ is the observed agreement between raters, denoted by the accuracy, while $P_{e}$ is the expected accuracy, represented by the chance agreement.

**Figure 2:**
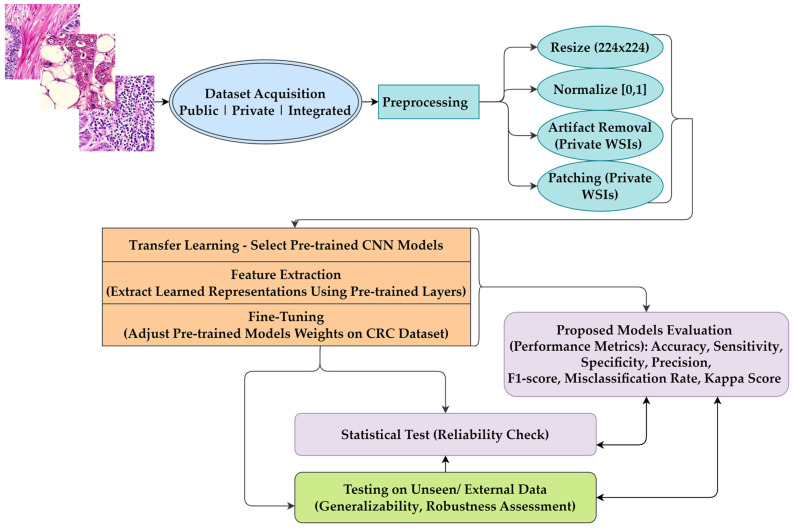
Overview of the proposed methods for classifying CRC histopathological images.

As detailed in Table 2 and illustrated in Figure 2, the proposed models begin by leveraging pre-trained models, which are fine-tuned for classifying CRC images. To adapt the pre-trained models, we employed global average pooling to reduce the feature maps to a single feature vector for the dense layer. A dropout layer is incorporated to combat overfitting, while batch normalization is applied to stabilize and accelerate the training process, improving the models’ generalization capability on the CRC classification task. Each model employs a distinct configuration tailored to enhance the performance of CRC histopathological image classification, as illustrated in Table 3. The hyperparameter settings for all proposed models included an initial learning rate of 1 × 10^−4^ with the Adam optimizer [44], a categorical cross-entropy loss function, a batch size of 32, and training for 20 epochs, with early stopping to mitigate overfitting.

In our study, we introduce layer-depth fine-tuning, tailored for CRC histopathological image classification. Our approach evaluates the impact of tuning different portions of each model architecture. The proposed method includes (i) a controlled scheme for selectively unfreezing layer blocks within pre-trained models, (ii) a comprehensive evaluation using repeated statistical tests over multiple randomized runs to ensure performance consistency, and (iii) validation across internal, external, and integrated datasets to assess generalization under domain shift. We integrate interpretability through Grad-CAM visualizations and expert pathologist review to confirm that model attention aligns with clinically relevant regions. This approach contributes to a reproducible and domain-adapted strategy for improving model adaptation in CRC histopathology datasets.

## 6. Results and Discussion

To address our research objective, we applied a fine-tuning protocol across several CNN architectures. Rather than relying on ad hoc training, our approach varied the number and depth of unfrozen layers in a structured, layer-wise manner while standardizing optimizer settings, learning rates, and training epochs. The design isolates the effect of fine-tuning depth on model performance, enabling a controlled investigation into how different levels of representational adaptation impact CRC histopathological image classification. Furthermore, by applying such a strategy across internal, external, and integrated datasets, we ensured that observed improvements are attributable to effective feature transfer rather than dataset-specific fitting, an essential consideration in medical image analysis under domain shift.

In our study, we assessed the performance of the baseline (DenseNet121, InceptionV3, and Xception) and fine-tuned models (CRCHistoDense, CRCHistoIncep, and CRCHoistXcep) on CRC datasets, comparing the average F1-scores across these models to evaluate their effectiveness in classifying CRC histopathological images. We selected the F1-score for its balance between precision and recall, which is vital in CRC image classification, as it reduces both missed diagnoses and unnecessary treatments. Fine-tuning consistently improved F1-scores across datasets, with statistically significant gains confirmed by a paired Wilcoxon signed-rank test, yielding a *p*-value of 0.018, indicating a statistically considerable enhancement between the baseline and fine-tuned models. The proposed models achieved a significantly higher average F1-score (mean = 0.993, 95% confidence interval [0.990, 0.996]) than baseline models (mean = 0.986, 95% confidence interval [0.979, 0.993]); Wilcoxon W = 28.0, *p* = 0.018, with a large effect size. The result confirms that fine-tuning based on the model’s architecture characteristics is an effective strategy for improving model performance, thereby enhancing precision and recall in CRC histopathological image classification.

**Figure 3:**
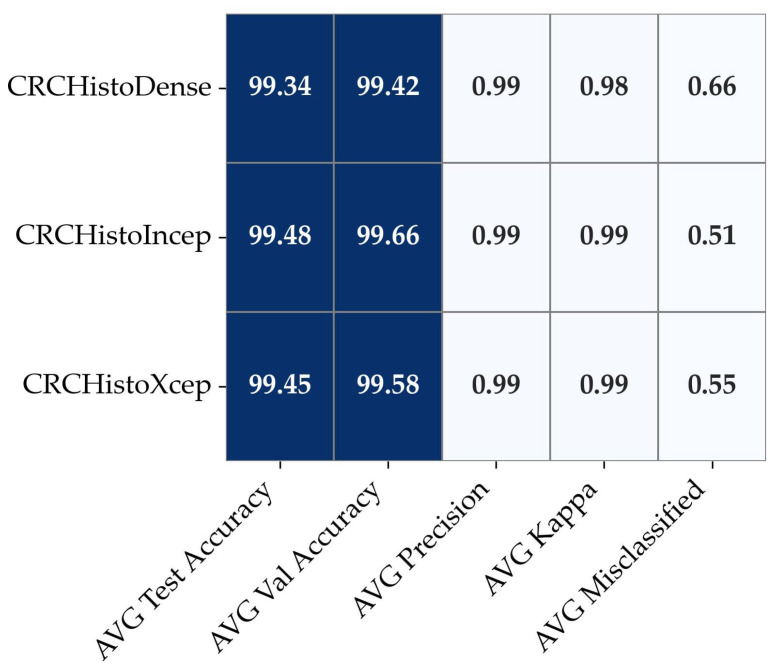
Heatmap of averaged performance metrics across proposed CRC models.

The fine-tuned models maintained consistently high performance across three distinct CRC histopathological datasets, reflecting their adaptability to data variation. As shown in Figure 3, the average classification metrics remained strong across all architectures, indicating that fine-tuning enabled effective feature extraction regardless of dataset source.

**Figure 4:**
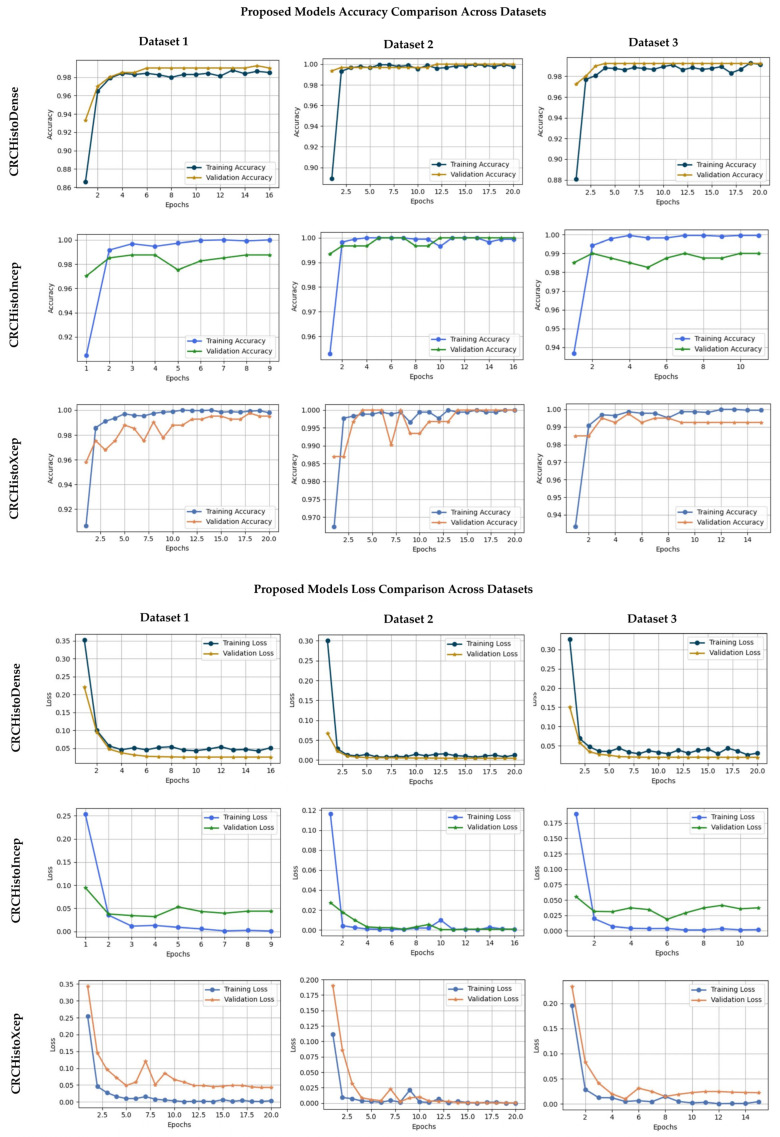
Accuracy and loss convergence across Datasets 1, 2, and 3 of the proposed models. The observed variations highlight differences in dataset complexity and reinforce the robustness of the fine-tuned models.

Figure 4 illustrates the training dynamics of the proposed models, including accuracy and loss curves, which highlight stable convergence and consistent generalization performance across different datasets.

Figure 4 shows the accuracy and the loss curves across private, public, and integrated datasets for the proposed models. CRCHistoDense exhibited the most stable learning behavior, with consistent convergence and minimal fluctuations, indicating robust generalization across diverse data sources. Although CRCHistoXcep marginally surpassed CRCHistoIncep in final accuracy, both followed comparable convergence patterns. Notably, CRCHistoDense and CRCHistoXcep required more training epochs to reach convergence, reflecting a deeper representational adaptation through fine-tuning. The smooth decline in loss across all models further confirms the stability of training and consistency of optimization. These trends highlight the effectiveness of our fine-tuning strategy in achieving balanced generalization across diverse CRC datasets. Such integrated analysis is essential for rigorous assessment and informed decision-making in advanced deep learning applications.

To empirically assess the impact of depth fine-tuning on model performance in CRC histopathological image classification, we employed an experimental design in which each proposed architecture was fine-tuned at multiple, predefined layer depths. For each depth setting, training was repeated 15 times with random seeds to capture variance due to initialization and sampling effects. Classification performance was assessed using the test accuracy metric via repeated-measures ANOVA, which allowed for us to evaluate the impact of fine-tuning depth while accounting for intra-model variability. This approach confirmed statistically significant improvements (*p* < 0.05) with deeper tuning across all models, supporting our hypothesis that depth fine-tuning influences feature adaptation and generalization. CRCHistoDense showed notable performance when the last 50 layers were fine-tuned (*p* = 0.028), while CRCHistoIncep benefited most from fine-tuning the last 100 and 150 layers (*p* < 0.05). For CRCHistoXcep, improved performance was achieved when the last 20 layers were fine-tuned, with statistically significant results. These findings, consistently replicated over multiple randomized trials (Table 4), highlight the critical importance of model-specific fine-tuning depth selection as a key factor in stabilizing training dynamics and enhancing cross-domain generalization.

To evaluate the generalizability of the proposed fine-tuned models, we performed cross-dataset validation using external and unseen data sources (Table 5). In the dataset of “CRC-VAL-HE-7K”, model performance remained consistent across internal, external, and unseen splits, demonstrating strong generalization when exposed to datasets with similar underlying distributions. In contrast, when the private dataset (Dataset 1) was used as the primary evaluation source, the models exhibited a noticeable performance drop when tested on external (public) and unseen samples from the same private source (672 samples). This decline reflects the increased complexity and heterogeneity of real-world histopathological images. The Kruskal–Wallis [45] test confirmed that the differences among internal, external, and unseen sets were statistically significant (*p* = 0.003), with post hoc pairwise analysis showing meaningful drops from internal to both external and unseen data (*p* = 0.011). However, there was no notable difference between external and unseen groups (*p* = 0.652), suggesting consistent generalization behavior in the presence of data variability. These findings highlight the practical challenge of domain shift in medical imaging and the effectiveness of our depth fine-tuning strategy. Specifically, the private dataset was curated in collaboration with clinical experts to reflect real-world diagnostic conditions, capturing adipose, muscle, and lymph node tissues under consistent imaging and preparation protocols. In contrast, the public datasets, although covering the same tissue types, were collected from independent sources with differing specimen handling practices and image resolution settings. These differences resulted in morphological variability and structural heterogeneity across CRC datasets. The consistent performance of the proposed models across internal, external, and unseen datasets highlights their robustness to such domain shifts and supports the generalizability of the depth fine-tuning strategy. Furthermore, the private dataset’s greater diversity served as a robust benchmark, revealing that model performance is sensitive to the complexity of source data. This cross-dataset analysis confirms that the proposed models enhance reliability and generalizability in CRC histopathological classification. These models achieve strong internal performance and maintain statistically significant robustness across unseen and heterogeneous samples, a crucial requirement for actual clinical settings.

While the proposed models’ outcomes are promising, we recognize the importance of evaluating potential sources of bias. The high results suggest effective model performance but also require careful interpretation, given the risk of overfitting in deep networks, especially with relatively limited or imbalanced medical datasets. We addressed this concern through structured fine-tuning and testing on diverse datasets. Nonetheless, future work should validate performance on larger colorectal cancer datasets and explore model calibration to ensure reliability in clinical deployment. Overall, the proposed models significantly improve CRC image classification while maintaining robustness and adaptability.

A key goal of our study is to enhance the performance and interpretability of the proposed CRC classification models that leverage three core characteristics: (1) depth-specific fine-tuning across pre-trained architectures to optimize feature adaptation to histopathological patterns, guided by statistical significance testing; (2) dataset curation based on clinical relevance, incorporating diverse tissue types (adipose, muscle, lymph node) and informed by pathologist input to better reflect CRC progression; and (3) model interpretability integration using Grad-CAM, enabling visualization of discriminative regions and validating model attention against expert-annotated tissue features.

Most existing CRC classification studies adopt off-the-shelf models with minimal architectural modification and apply standard fine-tuning strategies, typically freezing early layers or fine-tuning the entire network, without explicitly analyzing the impact of tuning depth. Our approach diverges by introducing a depth-aware fine-tuning strategy that explores intermediate adaptation levels. We selectively unfroze layers across different model blocks to evaluate their specific contributions to feature alignment and cross-domain generalization, particularly in the presence of morphological variability across private, public, and integrated CRC datasets. Repeated ANOVA-based statistical evaluations guided this fine-grained strategy to quantify learning gains associated with each adaptation depth. We aligned the overall performance trends with Grad-CAM visualizations, which confirmed that the models with deeper tuning yielded more coherent and clinically relevant attention patterns. We further observed that greater adaptation depth was necessary to accommodate semantic shifts across CRC datasets, but this could be controlled effectively using targeted learning rates and early stopping. These insights demonstrate that structured depth-aware fine-tuning can improve both generalization and diagnostic relevance in CRC classification.

**Figure 5:**
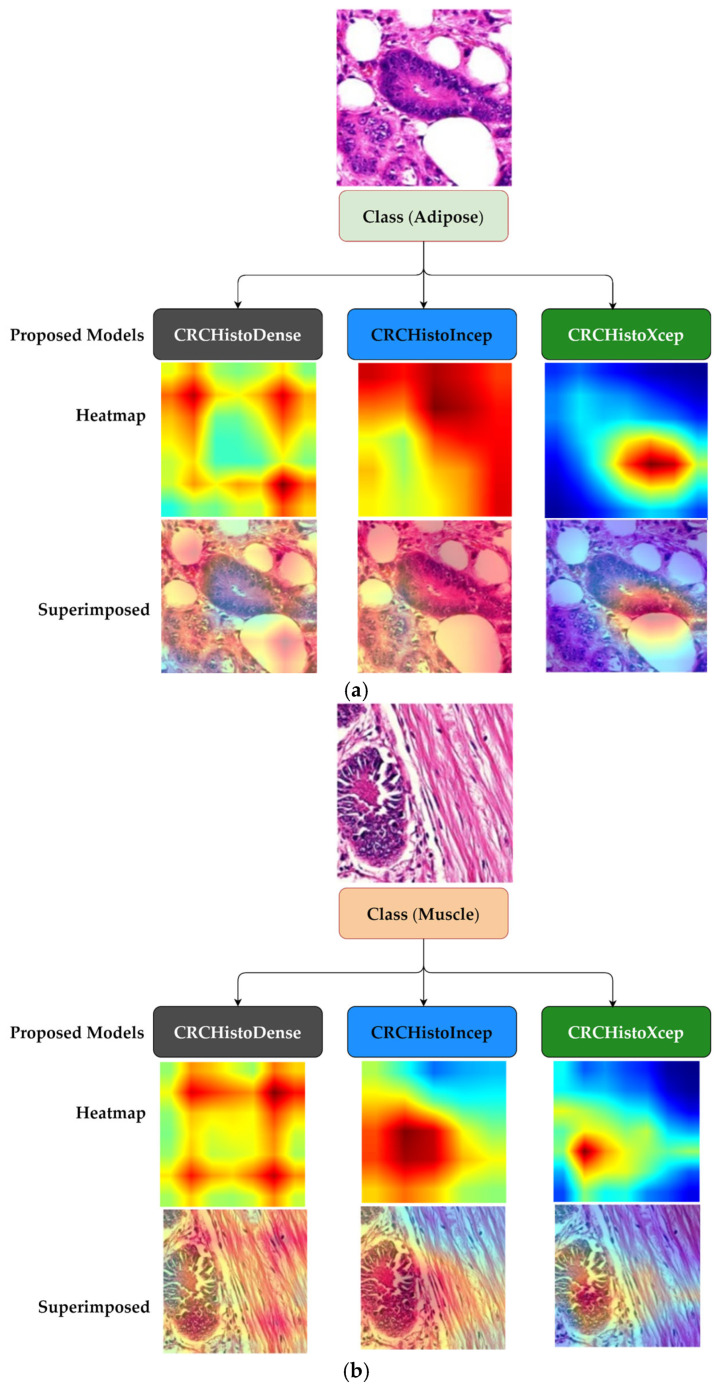

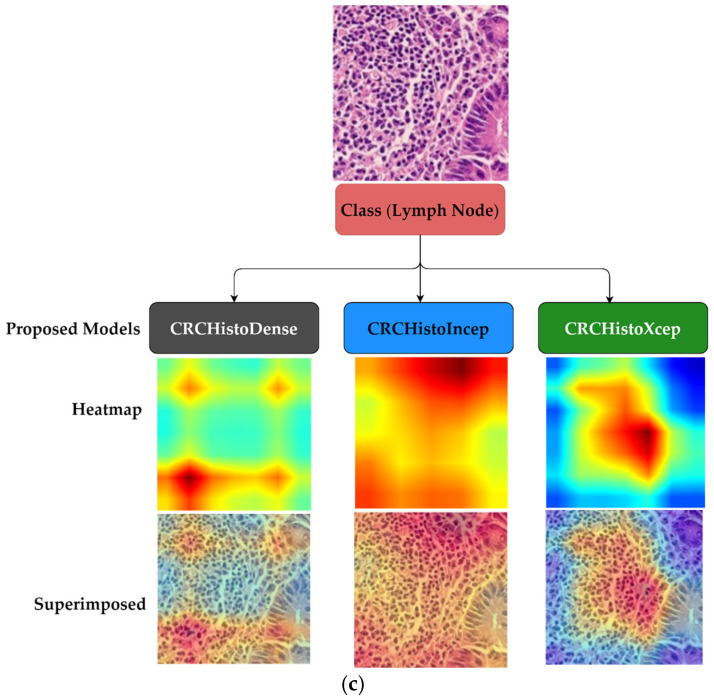
Visualization heatmaps highlight the regions identified by the proposed models as indicative of malignant and relevant features in CRC histopathological images for three classes: (**a**) adipose tissue, (**b**) muscle, and (**c**) lymph node.

Building on this and with the interpretability focus, Grad-CAM [46] visualizes the spatial focus of the proposed models during prediction. These visualizations provided insight into the regions influencing classification decisions, enabling us to assess whether performance improvements through fine-tuning were also accompanied by meaningful diagnostic reasoning (Figure 5c). To validate these interpretability outputs, we collaborated with pathologists who reviewed the heatmaps across different CRC classes. Their feedback confirmed that the highlighted regions corresponded to CRC histological findings, supporting the clinical coherence of the models’ attention. The validation confirms that the improved performance of the models is both statistically significant and clinically relevant. Our interpretability pipeline addresses transparency of deep learning models by providing visual justifications for the predictions of the proposed models and integrating clinical expert feedback. The outcomes provided evidence both technically sound and practically valuable, focusing on approaches that prioritize accuracy and offer explanatory depth.

To ensure the robustness and generalizability of our proposed fine-tuning strategy, we evaluated it across three widely used histopathology datasets (CRC5000, CRC7180, and LC25000). The objective of using these datasets was not simply to replicate single-dataset benchmarks but rather to demonstrate that our approach consistently improves performance across diverse data distributions and collection settings. In contrast to prior studies that relied on single datasets without addressing model transferability, our approach evaluated whether depth fine-tuning generalizes across internal, external, and heterogeneous datasets. This multi-dataset design enabled us to assess model stability and highlight dataset-specific challenges. Comparative results in Table 6 confirm that our proposed models achieve competitive performance, particularly when compared to studies using the same datasets, many of which lack explanations of the training protocol, statistical validation, or interpretability.

In the evolving landscape of deep learning in digital pathology, innovation does not rest solely on introducing novel architectures but also on effectively adapting and validating existing ones for actual clinical impact. Therefore, this study addresses that challenge by exploring depth fine-tuning strategies across established CNNs, DenseNet121, InceptionV3, and Xception, tailored for CRC histopathological image classification. While these models are not the newest in the field, their architectural stability, parameter efficiency, and well-characterized training behavior make them highly suitable for clinical tasks that require interpretability, generalizability, and reproducibility. Our objective was to investigate how depth fine-tuning and transfer learning strategies influence model performance and adaptability across various datasets. As highlighted by [47], who emphasized that fine-tuning strategies in histopathology remain underdeveloped, task adaptation has not yet been systematically explored. This study also raises the question of whether the transfer learning architectures optimized for natural images generalize effectively to medical domains. Our findings confirm that selectively fine-tuning, rather than full retraining or shallow transfer, consistently enhances classification accuracy and robustness, even across heterogeneous datasets with varied staining protocols and imaging conditions. To address concerns regarding model relevance, we also evaluated EfficientNetV2B0 under various fine-tuning configurations (50, 160, and 200 layers), including Adam (learning rate = 1 × 10^−4^), dropout (0.5), and dense (512). Despite its recent design, its performance (test accuracy ranges from 66.56% to 74.96%) was suboptimal across datasets. Resource limitations similarly hindered attempts to explore ConvNeXtTiny. As [48] emphasized, the success of deep learning in clinical applications depends not only on newer models but also on the ability to fine-tune existing ones efficiently and interpretably, especially in resource-constrained environments. Crucially, we integrated Grad-CAM-based visual interpretability, validated by expert pathologists, to ensure that the fine-tuned models not only performed well statistically but also localized diagnostically relevant regions. This interpretability validation reinforces the clinical reliability of our approach and addresses the gap between AI predictions and pathological decision-making, a step still lacking in many deep learning studies in histopathology.

We compared our models against prior studies using the same datasets. While a few works report slightly higher accuracy, many do not detail the fine-tuning strategies, optimization settings, or generalization capacity under domain shift. In contrast, our study contributes a structured and reproducible fine-tuning framework, validated statistically and tested across diverse CRC datasets. The analysis demonstrates that performance gains are critically shaped by how depth fine-tuning aligns with dataset variability. The observed consistency across internal, external, and unseen data, alongside clinically relevant interpretations, underscores the robustness and adaptability of our proposed configurations. These contributions offer valuable insights essential for advancing AI-assisted diagnostics in CRC histopathology.

## 7. Conclusions

This study examined the impact of fine-tuning depth on the performance of pre-trained models for CRC histopathology image classification. Through experiments conducted across multiple architectures, varied datasets, and statistically validated evaluations, we demonstrated that model-specific fine-tuning depth, which is dependent on the model’s architecture characteristics, can significantly enhance accuracy and generalizability. The integration of pathologist-guided dataset curation and model interpretability further reinforces the clinical relevance of the results. Our findings provide explicit fine-tuning strategies tailored to the model’s architecture for improving transfer learning in CRC classification tasks.

## Figures and Tables

**Table 1:** Distribution of CRC histopathological images across the three datasets, categorized by tissue classes (adipose, muscle, and lymph node).

Class	Dataset 1	Dataset 2	Dataset 3
Adipose	1014	1338	1073
Muscle	1239	592	1051
Lymph	1118	634	1210

**Table 2:** Fine-tuning approaches (CRCHisto) applied for CRC histopathological image classification.

1- Standard fine-tuning (baseline): The pre-trained model (M) is initially trained on a large source dataset (S). M = train (S) Proposed Model (PM) (Fine-tuning updates a subset of layers on the target dataset (T)). PM = fine-tune (M,T) Some layers are frozen (retain knowledge from S), and others are trainable (adapt to T). 2- Selecting the best layers to fine-tune: PM = fine-tune (M,T, selected layers) The selection depended on the model architecture and performance alignment with the CRC dataset features. 3- Statistical significance testing of PM: This test validated the impact of fine-tuning depth to ensure that *p* < 0.05. Statistically significant improvement. 4- External validation for generalization: Performance (PM, T) ≈ Performance (PM, unseen data) ≈ Performance (PM, external source) Tested the PM works beyond the training data on unseen and external datasets to validate it.

**Table 3:** Summary of the proposed models (CRCHisto)—fine-tuning approach. **: exponentiation.

Configurations	CRCHistoDense	CRCHistoIncep	CRCHistoXcep
Fine-tuned Layers	Last 20, 50, 75 layers	Last 25, 100, 150 layers	Last 20, 36, 50 layers. Layers from block8_speconv1 onward
Newly Added Layers	Dense (512)	Dense (1024)	Dense (1024)
Dropout Rate	0.5	0.5	0.5
Batch Normalization	Yes	No	Yes
Learning Rate (LR)	Schedule 1: If epoch < 10, then: LR = 1 × 10^−4^ × (0.1 ** (epoch//3) Else: LR = 1 × 10^−4^ × (0.1 ** (10//3)) Schedule 2: LR = 1 × 10^−4^ × (0.1 ** (epoch//%))	If epoch < 10, then: LR = LR Else: LR = LR × exp (−0.1)	Fixed LR = 1 × 10^−4^/ If epoch < 10, then: LR = LR Else: LR = LR × exp (−0.1)
Other Configurations	Early Stopping: Monitor: validation loss Patience: 3, 5 epochs restore_best_weight: True. Reduce LR on Plateau: monitor = validation loss, factor = 0.1, patience = 3	Early Stopping: Monitor: validation loss Patience: 5 epochs restore_best_weight: True. Reduce LR on Plateau: monitor = validation loss, factor = 0.2, patience = 3	Early Stopping: Monitor: validation loss Patience: 10 epochs restore_best_weight: True. Reduce LR on Plateau: monitor = validation loss, factor = 0.1, patience = 5

**Table 4:** Test accuracy of the proposed models at fine-tuning levels and initialization variations.

Seeds/ Fine-Tuning Level	0	1	2	3	5	7	11	13	17	19	23	29	31	37	41
CRCHistoDense Model															
L20	98.9	99.2	98.9	99.1	98.9	99.1	99.1	99.5	99.1	98.9	99.4	98.8	99.2	99.1	98.9
L50	99.2	99.4	99.1	99.4	98.9	99.1	99.1	99.5	99.1	98.9	99.4	98.6	99.5	99.2	99.2
L75	99.1	99.2	98.6	98.9	98.9	99.1	98.9	99.7	98.9	98.8	99.4	98.9	98.9	99.2	99.1
CRCHistoIncep Model															
L25	98.6	98.3	98.5	98.2	98.3	98.5	98.5	98.6	98.8	98.6	98.6	98.3	98.5	98.5	98.6
L100	98.8	98.2	98.5	98.6	98.9	98.9	98.8	98.8	98.8	98.5	98.6	98.8	98.9	99.2	98.8
L150	99.4	99.1	99.1	99.2	99.4	99.2	99.5	99.5	99.1	98.8	99.1	99.2	99.1	99.1	98.9
CRCHistoXcep Model															
L20	99.5	98.8	98.8	99.2	99.5	98.8	99.1	99.1	98.9	99.1	98.6	99.1	99.4	98.9	98.9
L36	98.6	98.6	98.6	98.9	98.3	98.8	98.6	98.8	98.8	98.5	98.3	98.9	98.8	98.5	98.5
L50	99.2	98.6	98.6	98.8	98.3	98.9	98.9	98.3	98.8	98.6	98.5	98.8	98.9	98.6	98.6

**Table 5:** Evaluation of the proposed models on various dataset sources.

Proposed Model	Trained Dataset	Internal Dataset Accuracy %	Unseen Dataset Accuracy %	External Source Accuracy %	Accuracy Variation % (Unseen/External)
CRCHistoDense	Dataset1	98.96	90.67	82.07	−8.29/−16.89
CRCHistoIncep		98.51	91.56	91.56	−6.95/−6.95
CRCHistoXcep		98.96	91.85	82.52	−7.11/−16.44
CRCHistoDense	Dataset2	99.80	97.93	94.54	−1.87/−5.26
CRCHistoIncep		100.0	97.78	98.05	−2.22/−1.95
CRCHistoXcep		100.0	98.37	95.52	−1.63/−4.48

**Table 6:** Comparison of proposed models with prior methods on benchmark CRC datasets.

Existing Study	Architecture Used	Dataset	Existing Study Accuracy (%)	Proposed Models Test Accuracy (%)
[21]	ResNet 50	CRC 5000	94.86	94.4–96.3
[22]	ResNet 50	CRC 7180	97.7	97.1–99.16
[23]	VGG19	CRC 5000	91.2	94.4–96.3
[25]	MobileNetV2	LC25000 (10,000)	99.67	99.7–100
[28]	VGG16, ResNet50, ResNet152	CRC 5000	96.16–98.38	94.4–96.3
[29]	ResNet50, Inception V3, VGG 16, VGG19, ResNet152V2	CRC 5000	70–89.87	94.4–96.3
[31]	ResNet34, EfficientNetB4	CRC 5000	99.8–99.97	94.4–96.3

## Data Availability

The public dataset used in this study is available at Zenodo (https://zenodo.org/records/1214456), (accessed on 7 November 2024). The study data can be obtained from the corresponding author upon reasonable request.

## Abbreviations

The following abbreviations are used in this manuscript:

CRC	Colorectal Cancer
AJCC	American Joint Committee on Cancer
TNM	Tumor Lymph Nodes Metastasis
WSI	Whole Slide Image
CNN	Convolutional Neural Network
CRCHisto	Colorectal Cancer Histopathology
